# Editorial: Impression management strategies and environmental cues as focal factors in food research

**DOI:** 10.3389/fnut.2023.1254856

**Published:** 2023-10-06

**Authors:** Tobias Otterbring, Agata Gasiorowska, Michał Folwarczny

**Affiliations:** ^1^University of Agder, Kristiansand, Norway; ^2^SWPS University of Social Sciences and Humanities, Wrocław, Poland; ^3^University of Galway, Galway, Ireland

**Keywords:** impression management, self-presentation, food science, sustainable consumption, retailing, consumer behavior, food choice, consumer psychology

Social interactions can be viewed as theatrical performances in which individuals play roles and display artifacts (1). Purposeful actions aimed at changing the way others perceive and treat an actor are also referred to as impression management (2), defined as “the process by which people control the impressions others form of them” [(3), p. 34]. Some consumption-related responses can be used as impression management tactics, because consumers purchase products not only for their immediate benefits, but also because certain purchases can help buyers either to stand out from the crowd or for others to perceive them as more desirable on specific traits, attributes, and personal characteristics (4–6). This idea also applies to food-related decision-making. For example, people sometimes order dishes they do not usually eat or eat differently to convey a positive image of themselves (7). Recent literature further suggests that sustainable food choices may act as an impression management tactic to increase perceived social standing, facilitate cooperation, and attract romantic partners [for a review, see Folwarczny et al. (8)].

This Research Topic focuses on various impression management strategies and environmental cues that can trigger cognitive, attitudinal, and behavioral changes related to food consumption. The Research Topic not only delineates and exemplifies how consumers strategically shift their food preferences based on contextual cues and situation-specific circumstances but also highlights how retailers can modify their shopping environments to create positive perceptions in shoppers. The included articles combine a variety of methods, such as Google Trends analyses, laboratory experiments, and analyses of real retail data. As such, the Research Topic combines the rigor and control characterizing wisely conducted laboratory experiments with new technology and the ecological validity of studies conducted “in the wild,” as called for by several scholars [e.g., Otterbring et al. (9)]. Together, the four articles described below contribute to research on impression management strategies in food-related decision-making contexts, particularly in the areas of health and sustainability.

First, Leary et al. combine research on goal conflict theory with research on masculinity stress to test and provide empirical support for the thesis that once men who experience masculinity stress have managed to signal their masculinity through certain “manly” products, they no longer have the motivation to continue making “macho” decisions. Accordingly, they can then form preferences even for products with a feminine connotation, like plant-based meat alternatives, although such products initially represent inhibited goals. Leary et al. use Google Trends data to demonstrate that masculinity stress, captured through specific search terms (e.g., erectile dysfunction, penis enlargement, how to get girls), is positively correlated with consumer search behavior for red meat and is negatively correlated with search behavior for plant-based meat alternatives. Their second study then shows that men, but not women, who experience masculinity stress are more prone to prefer and choose plant-based meat alternatives if such alternatives are available within a masculine context. Lastly, their third study documents that ethical considerations rather than masculine goals serve as a mechanism that guides men's preferences for plant-based meat alternatives when under the influence of masculinity stress.

Second, Ljusic et al. examine the role of technology-enabled healthy food labels on online grocery shopping among more and less impulsive customers. In a three-stage conjoint experiment, the authors consider three technology-enabled healthy food labels derived from research that theoretically should be linked to self-control. Participants initially have their impulsivity measured, and three distinct technology-enabled food labels (i.e., self-monitoring, pre-commitment, and social comparison) are then examined in relation to food choices, followed by participants providing demographic details. Ljusic et al. find that technology-enabled healthy food labels based on self-monitoring have the greatest impact on food choices, and that labels based on social comparison have the least impact, with labels based on pre-commitment falling between these two extremes. However, self-monitoring and pre-commitment labels have a greater impact on food choices for more (vs. less) impulsive participants, whereas social comparison labels more strongly influence food choices among less (vs. more) impulsive participants.

Third, Larsen et al. describe how retailers can use impression management strategies and environmental cues inside their stores to influence customers' shopping behavior toward healthy food options. The authors discuss three shopping-relevant categories—reaching, stopping/holding, and closing a sale—as conversion tactics that can be used by retailers to foster healthier food choices among customers. They then test the effects of placing healthy (vs. unhealthy) food products on a floor display in the store with the most traffic, with (vs. without) background music and an advertisement. According to the findings, sales of healthy food products can be higher than sales of popular unhealthy food products if promoted and positioned properly, as demonstrated by the floor display of a targeted healthy product.

Finally, Gasiorowska et al. examine whether and under what conditions consumers are increasingly inclined to prefer gender-typical food products as an impression management tactic. Based on impression management theories and literature on sex differences in human mate preferences, the authors experimentally test whether consumers' preferences for masculine or feminine foods are situation-specific and hence contingent on how the food consumption setting is framed: dining with an attractive date (mating) or eating out with friends (non-mating). Consistent with their conceptualization, they find that females generally express a stronger urge to consume “feminine” foods (e.g., seafood, salad), whereas males are more motivated to consume “masculine” foods (e.g., streak, burger). Moreover, females imagining a dinner with an attractive date—but not females imagining meeting and eating with friends—show a particularly powerful preference for such feminine foods. Interestingly, males prefer more “masculine” foods when imagining dining out with friends, but not when imagining dining with an attractive date, thus challenging one of the authors' key hypotheses and some prior findings in the literature.

We hope these articles shed new light on impression management strategies in various drinking and dining settings, and we encourage further research on such strategies within and beyond the food domain.

## Author contributions

TO: Writing—original draft, Writing—review and editing. AG: Writing—original draft, Writing—review and editing. MF: Writing—original draft, Writing—review and editing.
